# Integrated miRNA expression dataset in ruminant hosts naturally infected with cystic echinococcosis

**DOI:** 10.1016/j.dib.2026.113349

**Published:** 2026-10-12

**Authors:** Paola Pepe, Giovanna Maria Ventola, Elena Ciccone, Giovanna Marchese, Nunzio Antonio Cacciola, Antonio Bosco, Maria Paola Maurelli, Jacopo Guccione, Mariele De Santi, Gaetano Oliva, Laura Rinaldi

**Affiliations:** a Department of Health Sciences, University of Catanzaro Magna Gracia, Catanzaro, 88100, Italy; b Genomix4Life Srl, Baronissi, 84081, Italy; c Department of Veterinary Medicine and Animal Production, University of Naples Federico II, Naples, 80137, Italy; d Genome Research Center for Health-CRGS, Baronissi, 84081, Italy

**Keywords:** MicroRNAs, *Echinococcus granulosus sensu lato*, Protoscoleces, Germinal membranes, Ruminant, Bioinformatics analysis

## Abstract

Cystic echinococcosis is a zoonotic infection caused by the larval stage of *Echinococcus granulosus sensu lato* and remains endemic in several livestock-producing regions. This article describes a dataset of microRNA (miRNA) profiles obtained using *Echinococcus* cyst material collected from naturally infected sheep, cattle, and water buffalo in southern Italy, providing a valuable resource for the characterization and comparative analysis of miRNA profiles across different ruminant host species.

The dataset includes 38 samples obtained from different cyst matrices, including 10 protoscoleces (PSCs), 11 fertile and 17 sterile germinal membranes. Total RNA was extracted and processed using high-throughput sequencing technology. Raw sequence data, quality-filtered reads, and annotated miRNA entries are provided. A total of 168 mature molecules were identified using standardized bioinformatics workflows, including quality control, adapter removal, sequence alignment, annotation against reference databases, normalization procedures, and differential expression analysis. Raw fastq files are publicly available in the Sequence Read Archive under accession SUB16008388.

Supplementary Tables provide raw and normalized counts for each sample, together with information on host species, cyst localization, biological matrix, fertility status, and sample quality parameters. Sequencing metrics include raw reads, retained reads following processing and adapter trimming, percentage of reads retained, and the number of mature miRNAs detected. Differential expression results are also provided for fertile vs sterile membranes, PSCs vs fertile membranes and PSCs vs sterile membranes.

Overall, this dataset provides a comprehensive small RNA-Seq resource supporting studies of parasite developmental biology, host-associated variation, molecular marker discovery, comparative omics analyses, and the development of bioinformatics workflows.

Specifications Table

**Table:** 

Subject	Biology
Specific subject area	Molecular parasitology; small non-coding RNA profiling
Type of data	miRNA-Seq raw data (FASTQ format), Raw read counts (.csv format), Normalized counts (.csv format), Sample information (.csv format), sequencing matrix (.cvs format), Different expression analysis data (.csv format), Table (word format) Figures (jpg)
Data collection	Indexed libraries were prepared from purified RNA using the TruSeq SmallRNA Sample Prep Kit (Illumina). Libraries were quantified using the Tape Station 2200 (Agilent Technologies) and the Qubit Fluorometer 1.0 (Invitrogen Co., Carlsbad, CA). The identification and quantification of miRNAs was performed using the library mode of sRNAbench using miRBase: egr (release 22.1) as reference library. Using R software, the normalization was performed, applying negative binomial generalized linear models and retaining miRNAs expressed with at least a sum of 10 reads across all samples. Differential expression analysis was conducted between several groups using the Bioconductor package DESeq2 in R
Data source location	Department of Veterinary Medicine and Animal Production, University of Naples Federico II, Napoli, Campania Region, Italy
Data accessibility	Repository name: Expression profiles of miRNAs in ruminant intermediate hosts with Cystic echinococcosis, Feb 16 '26 Data identification number: SRA: SUB16008388 - BioProject: PRJNA1424680 Direct URL to data: https://www.ncbi.nlm.nih.gov/bioproject/?term=PRJNA1424680 https://dataview.ncbi.nlm.nih.gov/?search=SUB16008388&archive=biosample
Related research article	Expression profiles of miRNAs in ruminant intermediate hosts with Cystic echinococcosis. Ciccone, E., Pepe, P., Ventola, G. M., Marchese, G., Cacciola, N. A., Bosco, A., Maurelli, M. P., Guccione, J., De Santi, M., Oliva, G., & Rinaldi, L. (2026). Veterinary Parasitology (https://doi.org/10.1016/j.vetpar.2026.110853)

## 1 Value of the Data

• These data provide a comprehensive collection of miRNA profiles derived from *Echinococcus* cyst material isolated from naturally infected sheep, cattle, and water buffalo and represent a resource for researchers studying *Echinococcus granulosus s.l.* developmental biology and host-parasite interactions.
• The comparison of miRNA profiles between protoscoleces and germinal membranes can be used to identify candidate regulatory molecules and novel molecular targets involved in the biological cycle of *Echinococcus granulosus s.l.*, providing insights for the development of strategies to reduce parasite transmission.
• The analysis of miRNA expression across different intermediate host species (sheep, cattle, and water buffalo) can be used to investigate host-associated differences in *Echinococcus granulosus s.l.*, supporting studies on host specificity and parasite adaptation.
• The dataset can support the identification of novel parasite-derived biomarkers for the early diagnosis of cystic echinococcosis in intermediate hosts.
• The normalized read counts, raw reads, and annotation tables can be used to develop and benchmark small RNA-seq analysis pipelines for other helminths and to re-analyze the data as updated *Echinococcus* miRNA annotations become available.
• The dataset also enables meta-analyses integrating these profiles with other cestode or trematode small RNA datasets, supporting comparative genomics studies of miRNA evolution and function across parasitic flatworms.

## 2 Background

High-throughput sequencing approaches are increasingly used to characterize small non-coding RNAs in parasite-derived materials and to generate standardized transcriptomic resources. In this context, the present dataset was developed to investigate the microRNA (miRNA) profile of Cystic echinococcosis (CE) across different developmental stages of the zoonotic cestode *Echinococcus granulosus sensu lato* (*s.l.*) and across three intermediate host species: sheep, cattle, and water buffalo. The aim was to improve knowledge of the molecular biology of *E. granulosus s.l.* and identify novel molecular targets for the diagnosis and control of this still highly endemic parasitosis [1], particularly in Mediterranean regions [2,3]. The dataset provides structured access to miRNA sequencing data obtained using *Echinococcus* cyst materials, protoscoleces (PSCs) and germinal membranes, collected from naturally infected ruminant species (sheep, cattle, and water buffalo), enabling comparative investigations of miRNA profiles across host species and cyst developmental stages. This data article provides access to the complete sequencing output and processed files associated with the experimental workflow. It complements the related research article [4] by making the underlying dataset and supplementary bioinformatics resources available for reuse, reanalysis, and integration with other molecular datasets.

## 3 Data Description

The dataset was generated using different biological matrices, including PSCs and germinal membranes obtained from both fertile and sterile *Echinococcus* cysts collected at slaughterhouses in different ruminant intermediate hosts, namely sheep, cattle, and buffaloes. The number of samples used to generate the dataset, according to host species, biological matrix and cyst fertility status are summarized in Table 1.

**Table 1:** No. samples (PSCs, fertile and sterile germinal membranes) selected for each host species and used to generate the dataset.

Host species	Biological matrix	Cyst fertility status	No. of samples
Sheep	Protoscoleces	Fertile	6
	Germinal membrane	Fertile	7
	Germinal membrane	Sterile	2
*Sub-total, sheep*			**15**

Cattle	Germinal membrane	Sterile	10
*Sub-total, cattle*			**10**

Water buffalo	Protoscoleces	Fertile	4
	Germinal membrane	Fertile	4
	Germinal membrane	Sterile	5
*Sub-total, water buffalo*			**13**

Small RNA sequencing (smallRNA-Seq) experiments were performed on all samples using a single-end sequencing approach. Prior to downstream analyses, sequencing data quality was assessed through a Quality Control (QC) procedure aimed at evaluating the reliability and integrity of the raw sequencing reads. QC analyses were performed using the software FastQC [5], a widely used tool for high-throughput sequence data analysis. Quality evaluation was based on Phred quality scores. In our samples, the mean Phred quality scores ranged between 25 and 36, indicating high sequencing quality, as shown in Fig. 1. Furthermore, the per-sequence quality score distribution showed a predominant Phred score of 36 across the reads, confirming the overall excellent quality of the sequencing data (Fig. 2).

**Fig. 1:**
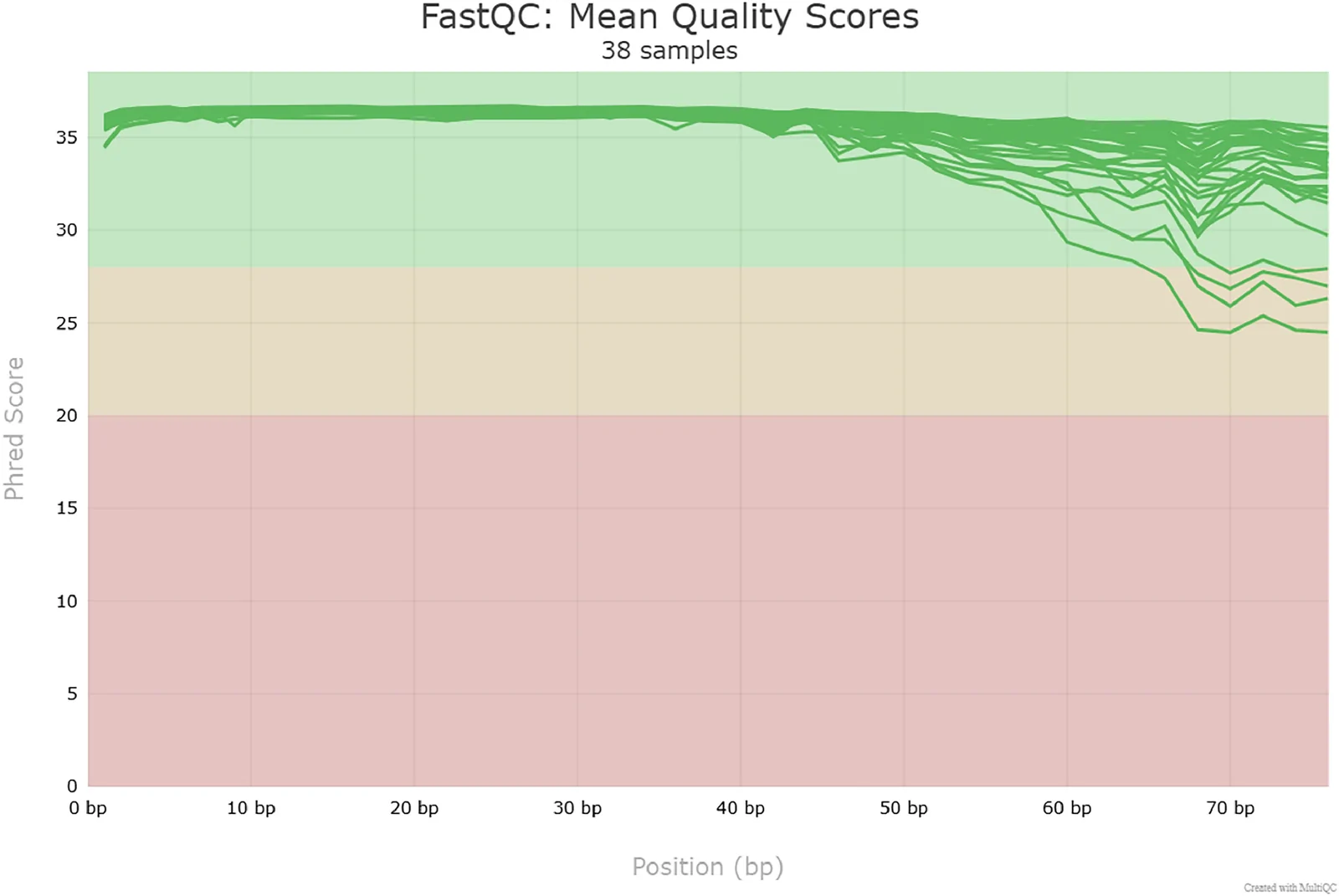
FastQC mean quality score distribution of the smallRNA-Seq dataset. The plot shows Phred quality scores ranging between 25 and 36 across the sequenced reads, indicating high overall sequencing quality and reliability of the generated data.

**Fig. 2:**
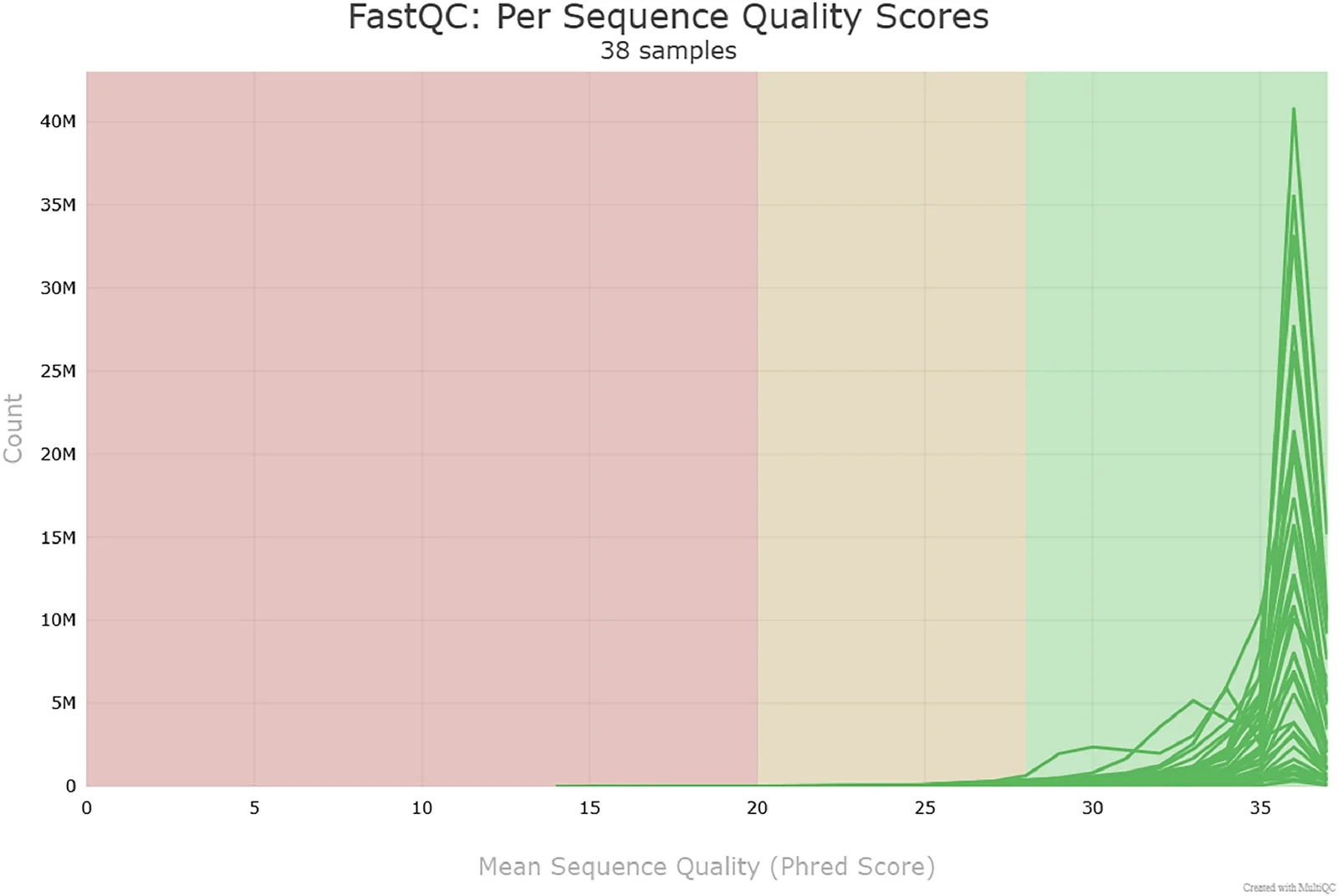
FastQC per-sequence quality score distribution of the smallRNA-Seq dataset. The graph shows that the Mean Sequence Quality Phred Score of reads is about 36, indicating excellent sequencing accuracy and overall high-quality reads.

In addition, adapter contamination and low-quality reads were addressed before further analyses. Adapter trimming and quality filtering were carried out using the bioinformatics pipeline implemented in sRNAbench (v. 2.0–7/2020) [6], selecting the Illumina TruSeq protocol settings for small RNA libraries. The 3′ adapter sequence TGGAATTCTCGGGTGCCAAGGG was specified, with a minimum adapter match length of 10 nucleotides and a maximum of one mismatch allowed. Recursive adapter trimming was disabled, and adapter detection was initiated at position 0 of each read. Identification and quantification of miRNAs was performed using the library mode of sRNAbench using miRBase:egr (*Echinococcus granulosus*) *-* release 22.1 as reference library [7]. The clean mapped data were subjected to differential expression analysis. The raw data (.fastq files) of 38 samples are available at Sequence Read Archive (SRA) under accession SUB16008388.

The dataset comprises raw sequencing data and processed files generated during the small RNA-seq analysis. The raw sequencing data are provided as FASTQ files and contain the sequencing reads generated for each sample. The processed data and associated sample information are provided in Supplementary Tables 1, which comprises several Excel sheets describing the raw and normalized miRNA read counts, sample metadata including host species, biological matrix, cyst fertility status and sample characteristics, as well as processing metrics for each sample. These metrics include the number of raw reads, reads retained after processing and adapter trimming, the percentage of reads retained for analysis, and the number of mature miRNAs detected. Differential expression results are provided in Supplementary Tables 2 and include the three pairwise comparisons: fertile versus sterile germinal membranes, PSCs versus fertile membranes, and PSCs versus sterile membranes. Sample identifiers are consistently used across the different files and tables, allowing the sequencing data, processed count matrices, metadata, sequencing metrics, and differential expression results to be linked and interpreted together.

## 4 Experimental Design, Materials and Methods

### 4.1 Sample collection, processing, and RNA extraction

A total of 101 *Echinococcus* cysts were collected from naturally infected sheep, cattle, and water buffaloes at slaughterhouses in Campania region, southern Italy, a highly endemic area for cystic echinococcosis. Detailed information on sample collection and analysis is provided in Ciccone et al [4]. Isolation of total RNA from samples (PSCs and germinal membranes from sterile and fertile cysts) was carried out using the QIAzol Lysis Reagent (QIAGEN, Hilden, Germany), following the manufacturer's instruction. Concentration and purity were measured using the NanoDrop instrument (Thermo Scientific™ Multiskan™ GO Microplate Spectrophotometer), and RNA integrity was assessed by 1% agarose gel electrophoresis. Among the samples collected, the ones showing degradation and/or not reaching a high concentration (> 50 ng/µl) were excluded for the subsequent analyses. Samples were then randomly selected to ensure an approximately balanced representation across host species and biological matrices. No fertile cysts were detected among cattle samples; consequently, PSCs and fertile membranes from cattle could not be included in the analyses (see Table 1).

### 4.2 Library preparation and sequencing

Indexed libraries were prepared from purified RNA using the TruSeq SmallRNA Sample Prep Kit (Illumina), following the manufacturer’s instructions. The pooled samples were subjected to cluster generation and sequencing, in a single run, using an Illumina NovaSeq 6000 System (Illumina) in a 1 × 75 single-end format. As all samples were processed and sequenced within the same experimental batch, no separate sequencing batches were present and batch effects related to sequencing runs were not applicable. The raw sequence files generated (.fastq files) underwent quality control analysis using FastQC.

### 4.3 Data analysis

After sequencing, demultiplexing was performed using the bcl2fastq tool (version v2.20.0.422 Copyright (c) 2007–2017 Illumina, Inc.) with the following parameters:–ignore-missing-bcls –ignore-missing-filter –barcode-mismatches 0 –no-lane-splitting -r 20 -p 20 -w 20 while all other settings were kept at default values. Quality assessment of FASTQ files was carried out using FastQC command-line tool [5] with the default parameters. Enriched miRNAs in each sample were identified using srnaBench pipeline, sRNAtoolbox Docker version (2022) Library Mod on the egr miRBase genome as a reference with the following parameter: microRNA=egr:oar protocol=I.

Differential expression analysis was performed using DESeq2 Bioconductor in R [8]. Before analysis, two filtering steps were applied: (i) features with zero counts across all samples were removed; and (ii) features were retained only if they showed non-zero expression values in >20% of the samples. The filtering was implemented using the following R codes: (i) matrix1=count[!(rowSums(count)==0),] # remove all zeros; (ii) matrix2= matrix1 [rowSums(matrix1!=0)>(ncol(matrix1)*0.20),] # retains miRNAs with non-zero values in >20% of the samples. All subsequent analyses were conducted according to the standard DESeq2 workflow and vignette recommendations (https://bioconductor.org/packages/release/bioc/vignettes/DESeq2/inst/doc/DESeq2.html#differential-expression-analysis). The differentially expressed miRNAs with adjusted *p-*value (padj) ≤ 0.05 and absolute (fold change) ≥ 1.5 were considered significant.

## Limitations

The limitation of this study is the still incomplete genomic annotation of miRNAs in the investigated species. Currently, only 218 mature miRNAs are available in public databases such as miRBase (https://www.mirbase.org/results/?organism=egr&chromosome=&start=&end), which may limit the comprehensive identification and characterization of miRNA expression profiles. Consequently, species-specific, low-abundance, or yet unannotated miRNAs potentially involved in the biological processes under investigation may remain undetected or poorly characterized. Moreover, reliance on the *E. granulosus* miRBase reference alone may have led to the omission of host-derived miRNAs present in the cyst material, as well as of novel or divergent parasite miRNAs not yet represented in the current annotation, potentially underestimating the complexity of the small RNA repertoire. An additional limitation arises from the natural-infection origin of the samples: since animals were not experimentally infected under controlled conditions, variability in infection duration, cyst fertility status, parasite strain, and host physiological factors could not be fully standardized, introducing a degree of biological heterogeneity that may have influenced the observed miRNA profiles. Finally, the relatively small sample size may reduce the statistical robustness of the analyses. Increasing the number of samples would improve the reliability and biological representativeness of the results.

## Ethics Statement

The authors confirm that they have read and comply with the ethical requirements for publication in data journals. The present work does not involve human participants, human data, animal experimentation, or data collected from social media platforms. Biological material was obtained from routine post-mortem inspections at a slaughterhouse, with no intervention on live animals.

## CRediT Author Statement

**Paola Pepe:** Investigation, Data curation, Formal analysis, Writing - Original draft. **Giovanna Maria Ventola:** Software, Formal analysis, Data curation, Writing - original draft. **Elena Ciccone:** Investigation, Data curation, Formal analysis, Writing - Original draft. **Giovanna Marchese:** Software, Validation, Data curation. **Nunzio Antonio Cacciola:** Resources, Investigation. **Antonio Bosco:** Resources, Investigation. **Maria Paola Maurelli:** Methodology, Investigation. **Jacopo Guccione:** Methodology, Investigation. **Mariele De Santi:** Visualization, Investigation. **Gaetano Oliva:** Methodology, Investigation. **Laura Rinaldi:** Conceptualization, Supervision, Funding acquisition, Project administration Writing – review & editing.

## Data Availability

SRA)SUB16008388 (Original data)

## Appendix Supplementary materials

Image, application 1

Image, application 2
